# Genetics of tolerance in honeybees to the neonicotinoid clothianidin

**DOI:** 10.1016/j.isci.2023.106084

**Published:** 2023-02-02

**Authors:** Nadejda Tsvetkov, Simran Bahia, Bernarda Calla, May R. Berenbaum, Amro Zayed

**Affiliations:** 1 Department of Biology, York University, Toronto, ON M3J 1P3, Canada; 2 Department of Entomology, University of Illinois at Urbana-Champaign, Urbana, IL 61801, USA

**Keywords:** Zoology, Molecular biology, Environmental toxicology

## Abstract

The effects of neonicotinoid insecticides (NNIs) on honeybee health are intensely debated, with numerous studies showing negative effects of exposure, while others report no such effects. We carried out experiments to study the genetic and molecular basis of NNI tolerance in honeybees, which may underlie the discrepancies observed in the literature. We discovered that worker survival post-exposure to an acute oral dose of clothianidin is heritable (*H*^2^ = 37.8%). Tolerance to clothianidin was not associated with differences in the expression of detoxification enzymes in our experiments. Instead, mutations in the primary neonicotinoid detoxification genes *CYP9Q1* and *CYP9Q3* were strongly associated with worker survival post-clothianidin exposure. In some instances, the strong association between CYP9Q haplotypes and worker survival was associated with the protein’s predicted binding affinity for clothianidin. Our findings have implications regarding future toxicological studies utilizing honeybees as a model pollinator.

## Highlights

• Bee survival after acute clothianidin exposure is heritable, with *H*^*2*^ = 0.38
• Gene expression in Malpighian Tubules of susceptible bees showed signs of apoptosis
• Non-synonymous mutations in *CYP9Q1* and *CYP9Q3* were associated with survival
• Models were used to calculate the proteins’ binding affinity for clothianidin

## Graphical abstract

**Figure:**
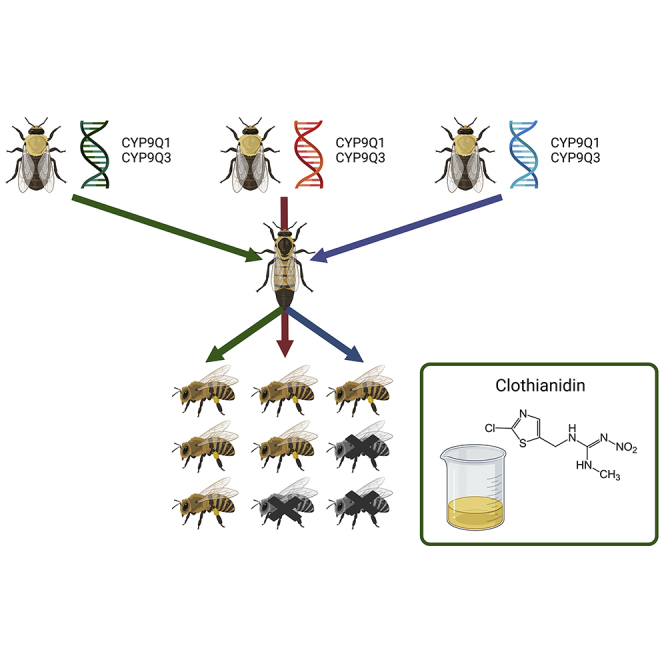

## Introduction

The relationship between neonicotinoid insecticides (NNIs) and the health of the western honeybee (*Apis mellifera*) is intensely debated.^1^^,^^2^^,^^3^^,^^4^ Many studies subjecting honeybees to sublethal NNI exposure documented a wide range of harmful effects,^5^^,^^6^ while others found no ill effects,^7^^,^^8^ especially at the colony level. Although some of these discrepancies can be attributed to the differences in study methodologies and parameters, including the dose and duration of NNI exposure,^2^ and environmental conditions such as local weather and colony health, there is some evidence that suggests that honeybee genotype may influence tolerance to NNIs. For example, there is substantive variation in the median lethal dose (LD_50_) estimated for NNIs in honeybees.^9^^,^^10^^,^^11^^,^^12^ The LD_50_ is defined as the dose at which 50% of the test animals die.^13^ Laurino et al.^14^ reported the acute oral toxicity at 24 h for the NNI clothianidin for five different colonies of *A. mellifera ligustica,* which ranged from 9.69 ppb to 41.96 ppb. In another study, Rinkevich et al.^12^ tested multiple colonies from genetically distinct honeybee subspecies^15^^,^^16^^,^^17^ and found that Italian honeybees (*Apis m. ligustica)* were 34 times more sensitive to the NNI imidacloprid than Carniolan bees (*A. m. carnica*).

Managed honeybee colonies have surprisingly high levels of genetic diversity owing to human-assisted migration and introgression,^18^^,^^19^ and some of this variation may underlie differences in NNI tolerance. While colony and subspecies differences in LD_50_ may suggest that NNI tolerance is heritable, they may simply reflect environmental differences between colonies (e.g., nutrition, exposure to other pesticides, and prevalence of disease). Controlled experiments are needed to determine if NNI tolerance is indeed heritable and the degree to which genetics contribute to this important trait. Addressing this knowledge gap is essential for understanding the impact of NNIs on honeybee health and for resolving conflicting experimental evidence on the acute and sublethal toxicity of NNIs to honeybees.

Resistance to NNIs has been studied in several pest insects. It is often modulated by increased metabolism of NNIs by cytochrome P450 monooxygenases (CYP) proteins, although resistance can also be caused by mutations in nicotinic acetylcholine receptors subunits, the target sites of NNIs.^20^ Enhanced metabolism by CYP proteins can occur through mutations that increase protein expression or amino acid changes in the CYP proteins that alter their structure in ways that can affect recognition, binding, and breakdown of the pesticides.^20^^,^^21^ In honeybees, the CYP3 clade is most closely associated with NNI detoxification.^22^ After testing all 27 genes in the CYP3 clade, Manjon et al.^23^ showed the NNIs are metabolized by members of the CYP9Q subfamily, primarily CYP9Q1, CYP9Q2, and CYP9Q3. Natural genetic variation in these genes may thus underlie variation in honeybee tolerance to NNIs.

In this study, we exploited patriline differences within honeybee colonies to estimate broad-sense heritablity of NNI tolerance. Honeybees are haplo-diploid and the queen is polyandrous,^24^ so relatedness between worker bees sired by the same father (i.e., supersisters) is 75%, on average, whereas that between workers sired by different fathers (i.e., half-sisters) is 25%, on average. Because all workers within a colony experience the same maternal effects and environment, partitioning of the phenotypic variance to within and between partlines within a colony can be used to estimate broad-sense heritability (*H*^*2*^)—the proportion of phenotypic variance that is influenced by genetic variance.^25^^,^^26^^,^^27^^,^^28^

We exposed individual worker bees from two colonies to the average LD_50_ dose of clothianidin^12^^,^^14^ and recorded whether bees survived or died after 24 h of exposure. We genotyped the workers from this experiment at many microsatellite loci and assigned them to patriline families. This framework allowed us to estimate the heritability of NNI tolerance, here defined as the proportion of a patriline that survived the clothianidin treatment at 24 h. To characterize the molecular mechanisms underlying NNI tolerance in honeybees, we carried out a second experiment on the same colonies and patrilines, in which we exposed individual workers to a sublethal dose of clothiandin.^5^ After genotyping and assignment to patrilines, we carried out transcriptional profiling to identify differentially expressed genes between patrilines with high or low tolerance to NNIs, as determined by the lethal dose experiment. Here we targeted three tissues where NNI detoxification enzymes are known to be expressed: the brain, the ventriculus (i.e., midgut), and the Malpighian tubules.^29^^,^^30^ We then sequenced portions of the *CYP9Q1*, *CYP9Q2,* and *CYP9Q3* genes from high- and low-survival patrilines and determined whether mutations in these genes segregated with the propensity of a patriline to survive the lethal dose at 24 h. Finally, we performed a molecular modeling analysis of some CYP9Q1 and CYP9Q3 haplotypes associated with survival in our experiment. Our analyses reveal new insights into the genetic and molecular mechanisms underlying NNI susceptibility in honeybees and their implications for using honeybees for toxicological testing of insecticides.

## Results

### Experiment 1: Patriline effect on mortality

We used a standard method to study the genetic contribution of worker traits using naturally multiply mated queens in social insects.^26^^,^^28^^,^^31^^,^^32^^,^^33^ We randomly picked two honeybee colonies for our experiment without any prior knowledge of their NNI tolerance to increase the generality of our findings, as commonly practiced in other patriline studies.^26^^,^^32^ We individually exposed 496 honeybee workers from two colonies, each with a naturally mated queen, to 29 ppb of clothianidin, the average oral LD_50_ value published for honeybees by the United States Environmental Protection Agency.^34^ The proportion of dead bees at 24 h post-exposure was 28% (69/247) for Colony 36 and 16% (40/249) for Colony 37, a statistically significant difference (Chi-squared test χ^2^ = 9.510, df = 1, p = 0.002). This lower than expected mortality (i.e., 50%) is consistent with the large degree of variability in NNI toxicity for different honeybee colonies and strains.^12^^,^^14^

**Figure 1:**
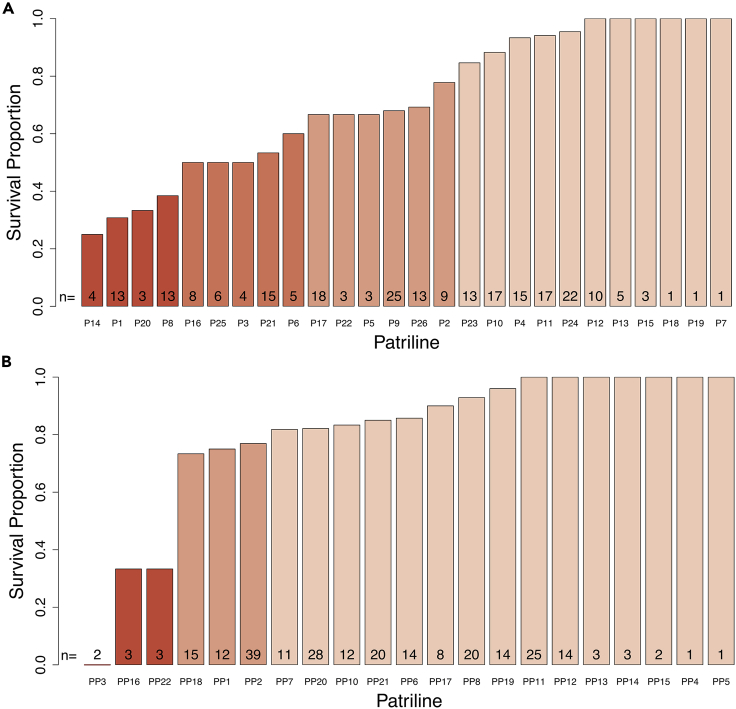
Patriline affects the survival of worker bees after clothianidin exposure (A) Colony 36 (Chi-squared test, χ^2^ = 57.842, df = 25, p < 0.001, N = 247) and (B) Colony 37 (Chi-squared test, χ^2^ = 35.387, df = 20, p = 0.029, N = 249). The numbers within each bar indicate the total number of workers tested for each of the patrilines. The height of the bar represents the mean.

We genotyped each worker at 11 hypervariable microsatellite loci to infer their father’s genotype and then grouped workers within a colony into different patrilines. We found 26 patrilines in Colony 36 and 21 patrilines in Colony 37—well within the range of published estimates of multiple mating for honeybee queens.^35^^,^^36^ In both colonies, patriline had a statistically significant effect on survival (Figure 1; C36: Chi-squared test, χ^2^ = 57.842, df = 25, p < 0.001; C37: Chi-squared test, χ^2^ = 35.387, df = 20, p = 0.029). Patriline also had a statistically significant effect on survival when both colonies were analyzed together (Analysis of deviance type II, LR χ^2^ = 98.769, df = 45, p < 0.001). The greatest amount of deviance was explained by the differences among workers within patrilines, with *D*^*2*^ = 0.791. The patriline component explained 18.9% of the deviance, leading to a *H*^*2*^ estimate of 37.8%. We repeated the analysis with patrilines that had at least five workers tested and found similar levels of deviance explained by each factor (Colony: 2.2%; Patrilines within the colony: 17.2%; Bees within patrilines within colonies: 80.6%; *H*^*2*^ = 34.4%).

### Experiment 2: Transcriptional profiling of neonicotinoid insecticide tolerant and non-tolerant patrilines

Experiment 1 demonstrated a genetic basis for NNI tolerance in honeybees. We carried out a second experiment on the same colonies to investigate transcriptional differences in three key tissues possibly associated with NNI tolerance in Experiment 1. We chose to focus on Colony 36, as it had the largest variation in bee survival. Worker bees (N = 367) were individually subjected to a sublethal dose of clothianidin (4.27 ppb). These bees were frozen 24 h post-exposure and were genotyped and assigned to the patriline whose survival was determined from Experiment 1. We chose three of the top surviving (“tolerant”) patrilines and three of the bottom surviving (“susceptible”) patrilines that had at least five bees each for further analysis. We dissected the brain, ventriculus, and Malpighian tubules of each bee and pooled each tissue from five workers per patriline. We compared gene expression profiles between the tolerant and susceptible patrilines to highlight genes that may be associated with NNI tolerance.

**Figure 2:**
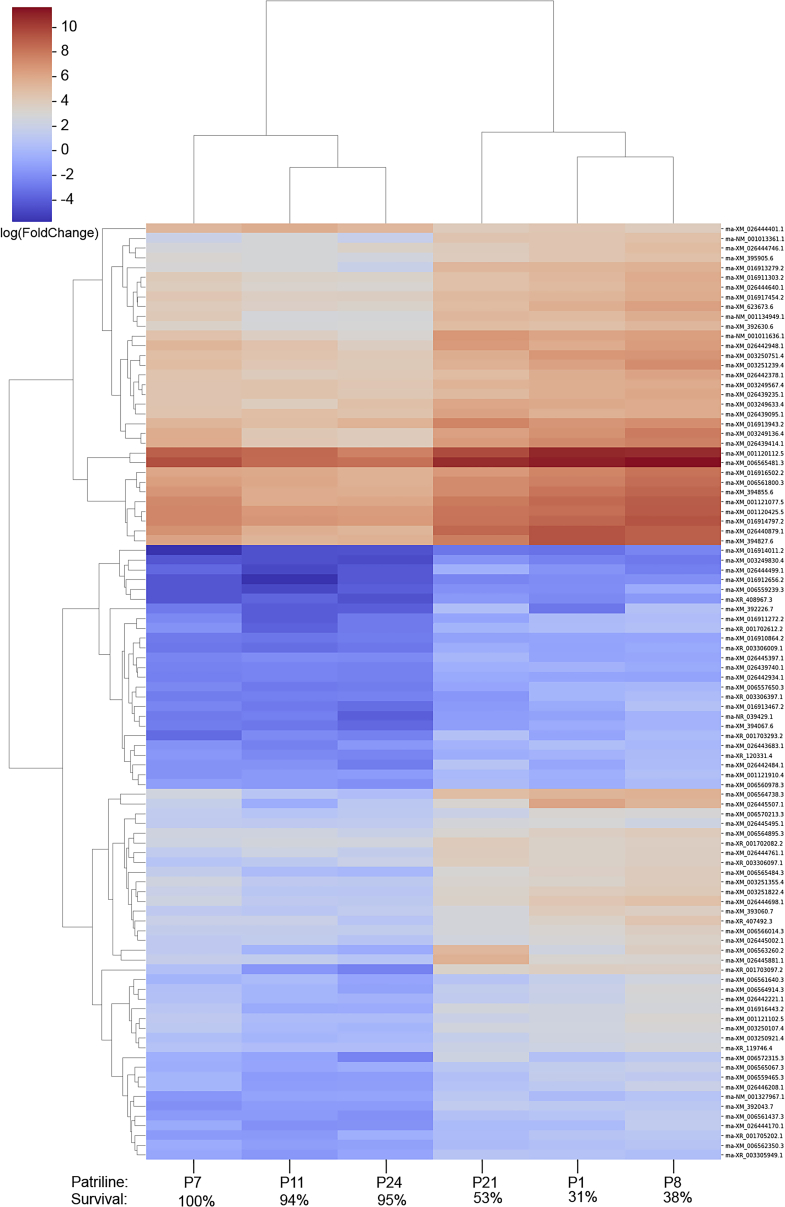
Gene expression differences associated with susceptibility to acute clothianidin exposure Heatmap of differentially expressed genes in the Malpighian tubules between tolerant and susceptible patrilines from Experiment 2. Survival denotes the average percent of workers that survived from Experiment 1.

We found no differentially expressed genes in the brain tissues of tolerant and susceptible patrilinies. We found a single gene, XR_003306097.1, encoding an uncharacterized protein that was upregulated in the ventriculus of susceptible bees (logFC = −3.35, p < 0.0001). The vast majority of transcriptional changes were observed in the Malpighian tubules (Figure 2). Tolerant patrilines had a single significantly upregulated gene in the Malpighian tubules: fatty acid amide hydrolase 2-B (logFC = 1.12, p < 0.0005). On the other hand, 95 genes were upregulated in the Malpighian tubules of susceptible patrilines (Sup. Table S1). These included Caspase-3, one of the main regulatory genes involved in apoptosis (logFC = −1.19, p < 0.05). Another gene, involved in osmotic regulation in the Malpighian tubules, the sodium-potassium calcium exchanger 3, was also upregulated (logFC = −1.50, p < 0.0005). Three SOX transcription factors were also upregulated in the susceptible group (Sox-2, logFC = −2.56, p < 0.0001; Sox- 1, logFC = −3.56, p < 0.001; and Sox 21-B, logFC = −1.71, p < 0.05). *CYP6A4* was the only cytochrome P450 among our differentially expressed genes; it was upregulated in the susceptible group (logFC = −1.47, p < 0.05).

Gene ontology analysis of the up-regulated genes in the Malpighian tubules of susceptible bees indicated proteolysis (GO:0006508) as the only enriched biological process (BP) (p < 0.016). This group includes endothelin-converting enzyme homolog, trypsin, disintegrin and metalloproteinase domain-containing protein 10-like, putative serine protease K12H4.7, caspase-3, aminopeptidase N (Gene ID: 551,180), aminopeptidase N (Gene ID: 551,224), and serine protease 53. Up-regulated genes in the Malpighian tubules of susceptible bees were also enriched for multiple molecular functions, including: peptidase activity (GO:0008233), peptidase activity-acting on L-amino acid peptides, beta-N-acetylhexosaminidase activity (GO:0004563), hydrolase activity (GO:0016787), hexosaminidase activity (GO:0015929), endopeptidase activity (GO:0004175), metallopeptidase activity (GO:0008237), catalytic activity, acting on a protein (GO:0140096), aminopeptidase activity (GO:0004177), catalytic activity (GO:0003824), serine-type peptidase activity (GO:0008236), and serine hydrolase activity (GO:0017171).

### CYP9Q mutations and neonicotinoid insecticide tolerance

To explore if genetic variation in key detoxification genes was associated with NNI tolerance, we sequenced the three CYP9Q genes encoding enzymes known to metabolize NNIs—CYP9Q1, CYP9Q2, and CYP9Q3^23^—in bees from Experiment 1. From each colony, the highest and lowest five patrilines in terms of survival with at least five tested bees were chosen for further analysis. We found non-synonymous mutations in all three CYP9Q genes (Figures S1–S3). Across all three genes, we identified 13 distinct CYP9Q haplotypes (i.e., 13 unique combinations of mutations across CYP9Q1, CYP9Q2, and CYP9Q3). These multigene CYP9Q haplotypes had a statistically significant effect on patriline mean survival (Analysis of deviance type II, Likelihood Ratio (LR) χ2 = 53.241, df = 14, p < 0.001). When analyzed individually, CYP9Q1 and CYP9Q3 haplotypes had a statistically significant effect on patriline survival (CYP9Q1: Analysis of deviance type II, LR χ2 = 49.610, df = 7, p < 0.001; CYP9Q3: Analysis of deviance type II, LR χ2 = 40.841, df = 8, p < 0.001). CYP9Q2 haplotypes did not have a statistically significant effect on survival (Analysis of deviance type II, LR χ2 = − 12.2188, df = 6, p = 1.00).

**Figure 3:**
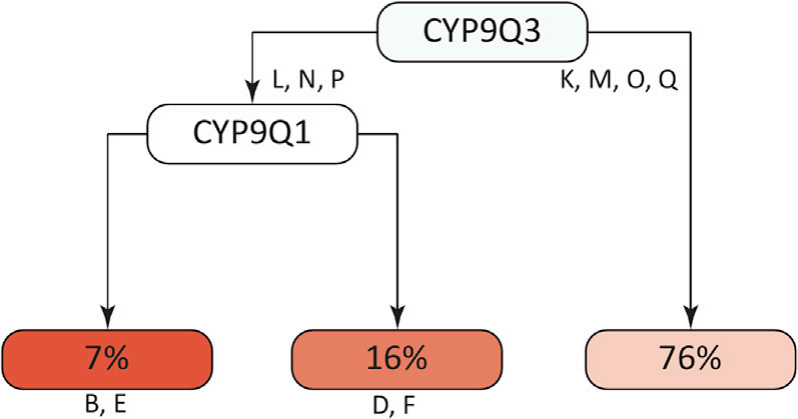
CYP9Q3 and CYP9Q1 haplotypes predict bee survival A classification tree analysis of the bee survival after acute clothianidin exposure by their CYP9Q1, CYP9Q2, and CYP9Q3 haplotypes. CYP9Q3 haplotypes had the largest effect on survival, while CYP9Q2 had no effect on survival. Letters denote the haplotype of the gene in the box directly above them. The percentage indicates the percent of bees that survived with those haplotypes.

We then carried out a classification tree analysis to examine how individual haplotypes at CYP9Q1 (n = 6), CYP9Q2 (n = 4), and CYP9Q3 (n = 7) were associated with NNI tolerance. The analysis revealed that CYP9Q3 and CYP9Q1 haplotypes best predicted survival (Figure 3; split = 2, CP = 0.01). CYP9Q3 appeared to have the largest effect on survival, with CYP9Q3 haplotypes L, N, or P associated with lower survival relative to other haplotypes (Figure 3). CYP9Q1 haplotypes modulated the effect of the survival-reducing haplotypes of CYP9Q3 (Figure 3). The combination of CYP9Q3 haplotypes L, N, or P and CYP9Q1 haplotype B or E had the lowest survival rates in our dataset. CYP9Q2 haplotypes did not improve survival prediction in the tree classification analysis.

### Predicted effects of individual mutations on the functionality of CYP9Q1 and CYP9Q3

The association between certain combinations of CYP9Q haplotypes and NNI tolerance suggests genetic interactions between CYP9Q genes are important for NNI detoxification. Accordingly, we examined the predicted effect of CYP9Q mutations based on modeling their ability to recognize, bind, and subsequently metabolize NNIs.

In CYP9Q1, there were five possible mutations found in the five haplotypes considered (Table 1: haplotype E was not considered as it produces a truncated protein). The mutation Gln60Glu was predicted to introduce a buried charge and thus potentially change the capacity to bind, recognize, or metabolize clothianidin; however, this residue is not part of any predicted SRS, catalytic site, or pocket. This mutation was found in haplotype A only, which had a high survival rate of 90%. The mutation Pro87Ser, present only in haplotype F, returned the highest score for mutational sensitivity in CYP9Q1 (Table 1); a Ser residue in that position is less favorable than Pro according to the resulting position-specific score matrix (PSSM), but the residue is not part of any predicted SRS, catalytic site, or pocket. This haplotype had a higher than average survival of 68%. The three other mutations in CYP9Q1 haplotypes (Ser213Ala, Met252Val, and Thr257Ala) all have relatively low mutational sensitivity scores (Table 1), but Ser213Ala is only two amino acids upstream of SRS2, hypothesized to be part of the substrate access channel in P450s. This mutation is present in two of the haplotypes B and D, which represent both high and low-surviving patrilines that segregate based on their CYP9Q3 haplotypes. Met252Val is located between SRS3 and helix K, and the Thr257Ala substitution is in helix K. SRS3 is part of the substrate access channel in P450s and helix K—identified by the ultra-conserved motif Glu-X-X-R in P450s—is in close proximity to the heme and believed to stabilize the structural catalytic core of P450s.^37^ These mutations are found in haplotype B that when present with CYP9Q3 haplotype N has a low survival rate, but when found with CYP9Q3 haplotype Q has a high survival rate. The Ser213Ala, Met252Val, and Thr257Ala are simultaneously present in haplotype B, indicating that this haplotype might have the most compromised functionality.

**Table 1:** Mutational sensitivity scores for the CYP9Q1 and CYP9Q3 haplotypes

Protein	Residue ID	Mutation	Haplotype	Score
CYP9Q1	60	GLN > GLU	A	16
	87	PRO > SER	F	85
	213	SER > ALA	B,D,E	22
	252	MET > VAL	B,E	8
	257	THR > ALA	B,E	5
CYP9Q3^a^	62	THR > MET	O,K,L,M,N	7
	152	THR > ILE	K	14
	238	GLY > ARG	N	6
	238	GLY > ASP	M	8
	288	MET>THR	L	11
	302	THR > −	P	NA^b^
	317	LEU > ILE	M,N	8
	380	VAL > ALA	M,N,L	12
	387	ALA > PRO	M,N	3

a CYP9Q3 Haplotype Q is identical to wild type, thus mutation score cannot be calculated. We did not calculate CYP9Q2 mutation scores.

b The algorithm does not score deletions; however, the average score for mutations in this site (to any of the other 20 amino acids) is the highest of all mutations found in our study.

In CYP9Q3, there were nine mutations identified across seven haplotypes, with several haplotypes having more than one mutation (Table 1). The mutational effect score of the substitutions is relatively low, except for the deletion of Thr302, which cannot be scored because Missense3D and SuSpect software only score substitutions. However, the average score of any possible substitution in Thr302 is 70, an average score in the highest 80th percentile across scores of all other residues in the protein (Table 1). Thr302 falls in helix I, involved in proton delivery and part of the core catalytic domain. Amino acid changes in helix I are rare as they would create distortions changing the interaction of the core catalytic site with both the heme and the substrate^38^; thus, the Thr302 deletion is predicted to be the most detrimental mutation among the ones detected. This deletion was present only in haplotype P, which had a survival rate of 68%. The mutations Gly238Arg/Asp in haplotypes M and N and Val380Ala in haplotypes M, N, and L could also interfere, to some extent, with the ability of CYP9Q3 to recognize, bind, and/or metabolize the substrate because they are located near SRS3 and SRS5. Haplotype M has a high survival rate of 75% and only appeared with a wild-type CYP9Q1 haplotype. Haplotype N had a low survival rate, regardless of the CYP9Q1 haplotype. Haplotype L had a survival rate of 51% and was only found with CYP9Q1 haplotype D.

### In-silico clothianidin docking with CYP9Q1 and CYP9Q3 variants

We used the crystal structure of CYP3A4 (PDB ID: 1TQN)^39^ as a template to build homology-based models for all the CYP9Q haplotypes using the MOE environment (Chemical Computing Group Inc., Montreal, Canada).

Docking of clothianidin in the predicted active pocket of each CYP9Q1 haplotype showed that haplotype C had the most favorable potential energy when docked. Haplotype C docked with clothianidin also had the shortest distance from its Thr308 in the I-helix to the heme. Haplotype A showed the second most favorable potential energy in the docked conformation, whereas haplotypes B, D, and F scored similarly in terms of final potential energy and distances from the heme oxygen intermediate to both the methyl group in clothianidin and the Thr308 residue in the P450.

Docking of clothianidin in the predicted active site of each CYP9Q3 haplotype showed that haplotype P would dock clothianidin with average final distances from the reactive site (methyl group in clothianidin) to the heme oxygen intermediate; these distances are likely too large (greater than 9 Å) to enable functionality (Table S2). These dockings also had the largest final interacting energy, indicating a less favorable interaction. The shortest distances between the methyl group in clothianidin and the heme as well as the lowest final interacting energy were found in haplotype O.

We found a statistically significant correlation between survival and final interacting energy of CYP9Q3 (Pearson’s product-moment correlation, t = −2.537, df = 18, p value = 0.021, r = −0.513). We also found a statistically significant correlation between survival and distance from the methyl group of clothianidin to the heme intermediate after E minimization for CYP9Q3, but only for the haplotypes from susceptible patrilines (Pearson’s product-moment correlation, t = 4.958, df = 3, p value = 0.016, r = 0.944). We found no statistically significant correlations between survival and the CYP9Q1 parameters (Pearson’s product-moment correlation, p > 0.05 for all parameters).

## Discussion

In this study, we investigated the degree to which NNI tolerance is heritable and whether gene expression or genetic variation in the CYP9Q1-3 genes can explain the difference in NNI tolerance. We first exposed nearly 500 honeybee workers to the average oral LD_50_ for the NNI clothianidin and tested whether workers surviving 24 h after exposure differed with respect to their patriline genetics from workers that died within 24 h of exposure. We found that patriline genetics had a strong and significant effect on 24 h survival after acute clothianidin exposure. The broad-sense heritability of this phenotype is 37.8%, suggesting a substantive genetic contribution to NNI tolerance in honeybees. While the environment clearly plays an important role in influencing tolerance to clothianidin, our results suggest that the large variation in LD_50_ estimated from different honeybee colonies and subspecies,^12^^,^^14^ and the large variation in “health” outcomes after NNI exposure in honeybees,^9^^,^^10^^,^^11^^,^^12^^,^^40^ may be partially influenced by honeybee genetics.

We explored two potential mechanisms underlying the heritability of NNI tolerance in honeybees. We first tested the hypothesis that tolerant honeybee patrilines express detoxification enzymes at higher levels than susceptible patrilines. We exposed bees from the same colonies used to demonstrate the heritability of NNI tolerance to a sublethal dose of clothianidin and collected them 24 h later for RNA sequencing. We reasoned that this sublethal clothianidin challenge would have allowed bees to activate their detoxification system, thereby allowing us to compare the expression level of cytochrome P450 genes in the tolerant versus susceptible patrilines. Our transcriptomic comparisons were independently carried out in three separate tissues known to be important for NNI detoxification in honeybees: the brain, ventricus, and the Malpighian tubules.^29^^,^^30^ Contrary to our predictions, we found no evidence of upregulation of CYP9Q1, CYP9Q2, CYP9Q3, or other P450s in worker bees from tolerant patrilines relative to susceptible patrilines. Moreover, GO terms associated with detoxification were not enriched among differentially expressed genes between these two groups. We found only a single cytochrome P450 gene, CYP6A4, among our differentially expressed genes, but its expression level was actually higher in the susceptible group. Our results here indicate that NNI tolerance is not mediated by higher expression of CYP9Q genes or other cytochrome P450 genes in tolerant patriline 24 h after exposure. It is possible that we missed the differences in the activation of the detoxification genes, if they occurred in less than 24 h. The majority of gene expression changes observed in the Malpighian tubules of susceptible patrilines post-exposure to clothianidin appear to be associated with clothianidin-induced damage to this organ as previously demonstrated.^41^^,^^42^

We then tested and found support for the hypothesis that allelic variations in CYP9Q genes underlie NNI tolerance in honeybees. We identified non-synonymous and presumably functional mutations in three CYP9Q genes. CYP9Q haplotypes were significantly associated with 24-h survival of bees exposed to clothianidin. Survival rate was particularly associated with CYP9Q3 haplotypes and—to a lesser extent—CYP9Q1 haplotypes. This finding is consistent with the findings of Manjon et al.,^23^ who showed that CYP9Q3 had the highest efficiency when metabolizing NNIs out of all of the CYP3 genes. Our analysis hints at complex epistatic interactions between CYP9Q3 and CYP9Q1 in modulating NNI tolerance, as CYP9Q1 haplotypes appear to modulate the main effect of CYP9Q3 haplotypes on survival. Epistatic effects of multiple mutations in detoxification genes have been reported previously.^43^^,^^44^ While Manjon et al.^23^ found that CYP9Q2 was a more efficient NNI metabolizer than CYP9Q1, CYP9Q2 haplotypes did not significantly affect survival in our experiment. This difference could be due to the similarity between the substituted amino acids we found in the CYP9Q2 haplotypes: Ile248Val and His467Tyr. Isoleucine and valine are both aliphatic and hydrophobic, while histidine and tyrosine are both aromatic and hydrophobic. These types of amino acid substitutions are unlikely to cause structural change in the protein.^45^^,^^46^ In other words, while CYP9Q2 plays an important role in metabolizing NNIs,^23^ the amino acid-changing mutations discovered herein were all conservative and unlikely to affect the structure and function of this protein.

Three other lines of evidence suggest that allelic variation in CYP9Q genes influences NNI tolerance in honeybees. First, at least one, and in some cases several, of the discovered non-synonymous mutations in CYP9Q3 and CYP9Q1 has a mutational effect score that is theoretically expected to affect how these proteins recognize, bind, and metabolize clothianidin. Second, at least one, and in some cases several, of the CYP9Q3 and CYP9Q1 haplotypes appear to compromise docking between the resulting proteins and clothianidin, based on in-silico docking analysis. Finally, there is a statistical association between some of the in-silico docking parameters with 24-h survival from Experiment 1; these results would be expected if mutations in CYP9Q genes were underlying the observed NNI tolerance in honeybees.

Our study demonstrated that honeybees have genetic variation that is statistically associated with the ability of worker bees to survive acute clothianidin exposure over the short term (24 h). The underlying mechanism of short-term NNI tolerance appears to be associated with allelic variants that compromise the ability of CYP9Q3 and CYP9Q1 to recognize, bind, and metabolize clothianidin, although additional functional work is required to validate this hypothesis. Our results are important for the field of ecotoxicology, where a small number of individuals are often used as a proxy for entire species and their guilds. For example, according to the United States Environmental Protection Agency, only 150 individual honeybees need to be tested to demonstrate the safety of a pesticide for the entire species, which may be from a single colony,^47^ and such a study can be used as a surrogate to infer the safety of the pesticide for “Terrestrial Beneficial Insects, Invertebrates” as a whole (EPA-HQ-OPPT-2009-0154-0028). Honeybees are managed by humans, and industrial beekeeping practices have enhanced gene flow and introgression among geographically and genetically distinct subspecies, resulting in managed bee populations that have greater levels of genetic diversity relative to their progenitors.^18^^,^^19^^,^^48^^,^^49^ A different genetic background could explain the discrepancy found in studies that exposed honeybees to a neonicotinoid and yielded conflicting results. Additionally, this raw genetic variation may allow honeybees to adapt to some emerging stressors in their environment. It is currently unclear if the CYP9Q mutations discovered herein also allow honeybees to tolerate other NNIs. It is also unclear if heritable tolerance to insecticides is common in honeybees. Nevertheless, our study clearly underscores the importance of considering genetics in the ecotoxicological assessment involving honeybees. To the extent that logistics permit, we strongly recommend that future ecotoxicological investigations maximize the number of colonies studied to provide more reliable toxicological inferences, especially in regulatory tests for pesticide risk assessment.

### Limitations of the study

One limitation of our study is that we only tested gene expression signatures associated with sublethal exposure in a single colony at a single time point after exposure. While we did not observe differences in the expression of CYP9Q1-3 genes 24 h after exposure to clothianidin between tolerant and susceptible patrilinies, such differences may have occurred prior to, or after we sampled our bees. Additionally, we tested one neonicotinoid and thus, we cannot conclude if heritability of tolerance exists for neonicotinoids in general or if the observed patterns in gene expression or genetic variations associated with mortality after exposure will be similar for other neonicotinoids. Finally, the computational work we performed on the binding properties of CYP9Q1 and CYP9Q3 is theoretical and requires validation using functional experiments.

## STAR★Methods

### Key resources table

**Table:** 

REAGENT or RESOURCE	SOURCE	IDENTIFIER
**Chemicals, peptides, and recombinant proteins**		

Clothianidin	Sigma-Aldrich	33589
Sucrose	Sigma-Aldrich	S0389
Taq 2X Master Mix	New England Biolabs	M0270L
Invitrogen™ RNAlater™	Fisher Scientific	AM7021

**Critical commercial assays**		

Mag-Bind® Blood & Tissue DNA HDQ 96 Kit	Omega Bio-Tek	M6399-01
miRNeasy Mini kit	Qiagen	217004

**Deposited data**		

Raw DNA data	This paper	GenBank: ON648906-ON649682
Raw RNA data	This paper	GEO: GSE216021

**Experimental models: Organisms/strains**		

*Apis mellifera*		

**Oligonucleotides**		

Primers for patrilines, see Table S3	This paper	N/A
Primers for CYP9Q1-3, see Table S4	This paper	N/A

**Software and algorithms**		

Geneious Prime	Kearse et al.^50^	https://www.geneious.com/
Phyre2	Kelley et al.^51^	http://www.sbg.bio.ic.ac.uk/∼phyre2/html/page.cgi?id=index
Catalytic Site Atlas 2.0	Furnham et al.^52^	https://www.ebi.ac.uk/thornton-srv/m-csa/
SuSPect software	Yates et al.^53^	http://www.sbg.bio.ic.ac.uk/suspect/
Missense3D	Ittisoponpisan et al.^54^	http://missense3d.bc.ic.ac.uk/missense3d/
Molecular Operating Environment (MOE)	Chemical Computing Group Inc., Montreal, Canada	https://www.chemcomp.com/Products.htm
Trimmomatic	Bolger et al.^55^	http://www.usadellab.org/cms/?page=trimmomatic
Spliced Transcripts Alignment to a Reference	Dobin et al.^56^	https://github.com/alexdobin/STAR
MultiQC	Ewels et al.^57^	https://multiqc.info/
HTSeq: High-throughput sequence analysis in Python	Anders et al.^58^	https://htseq.readthedocs.io/en/master/
Empirical Analysis of Digital Gene Expression Data in R	Robinson et al.^59^	https://bioconductor.org/packages/release/bioc/html/edgeR.html
R	Team^60^	https://www.r-project.org/
ShinyGO	GE et al.^61^	http://bioinformatics.sdstate.edu/go/
rpart: Recursive Partitioning and Regression Trees	Therneau et al.^62^	https://CRAN.R-project.org/package=rpart
rpart.plot	Milborrow^63^	https://cran.r-project.org/web/packages/rpart.plot/

### Resource availability

#### Lead contact

Further information and requests for resources and reagents should be directed to and will be fulfilled by the lead contact, Amro Zayed (zayed@yorku.ca).

#### Materials availability

This study did not generate new unique reagents.

### Experimental model and subject details

Honeybees for this study were obtained from two colonies with naturally mated queens located at York University (Toronto, ON, Canada). Like most North American colonies, these bees had a mixed genetic ancestry with major contributions from the East European population group (C group: *A. m. ligustica* and *A. m. carnica*) and minor contributions from the West European population group (M group: *A. m. mellifera*).^18^^,^^19^ Ready to emerge brood from two honeybee colonies were placed in a 33°C incubator. Every day, newly emerged worker bees were marked with a new color using non-toxic enamel paint (Testors) and introduced into their respective colonies. When the bees were seven days old, they were collected, placed into individual plastic Petri dishes with air holes, given a large 50% sucrose (Sigma-Aldrich) feeder; these dishes were then placed into a 33°C incubator overnight, separate from the brood.

### Method details

Eight days after emergence, the bees were randomly allocated into three groups: lethal dose , field dose (see below), and control. The lethal dose was 29 ppb of clothianidin (99.9%, Pestal, Sigma-Aldrich) representing the oral LD_50_ value obtained from the U.S. Environmental Protection Agency Pesticide Ecotoxicity database.^34^ The field dose was 4.27 ppb of clothianidin, which is the average clothianidin found in pollen in one field survey^5^ carried out in the same region as our study. The lethal dose was used to establish a clear phenotype for the genetic experiment, while the lower dose was used for the gene expression analysis (see below) since RNA degrades quickly after organism death. Clothianidin was first prepared using acetone and then diluted in 50% w/v sucrose solution, such that the final acetone concentration was 0.1%. The control group received 50% w/v sucrose solution containing 0.1% acetone.

We followed a standard protocol to test for acute oral toxicity,^47^ with the modification of testing one dose and each bee individually, as opposed to testing in groups. We also focused on 24-h mortality, as opposed to 48-h mortality, because we found no difference between 24 and 48 mortality in our pilot study (data not shown), which is in agreement with previous research.^14^ Briefly, the bees were starved for 2 h, after which they were given a pre-weighed small feeder with 20 μL of the appropriate sugar solution for 4 h, at which point the small feeder was removed, weighed, and replaced with a large feeder of 50% w/v sugar solution. These bees were then placed into the incubator and mortality was scored 24 h after the removal of the small feeder. All of the bees were frozen at −80°C. The mortality in the control group never exceeded 10% per 24 h. Bees that consumed less than 90% of the sugar solution were not included in the analysis. A total of 496 bees from the lethal dose group and 370 bees from the field dose group were used in subsequent analyses.

#### DNA extraction

DNA extraction was performed using the Mag-Bind Blood & Tissue DNA HDQ 96 Kit (Omega Bio-Tek) and the KingFisher Flex extraction system (ThermoFisher Scientific). Briefly, we cut the thorax in half and crushed it using a pestle, while being cooled in liquid nitrogen. Then, we added 350 μL of TL buffer and 20 μL of Proteinase K solution to the crushed thorax, vortexed for 10 s, and incubated at 50°C overnight. The next day, the sample was centrifuged for 10 min at 7000xg/rcf and 300 μL of the clear supernatant was transferred into an intermediate tube. The intermediate tube was centrifuged for 10 min at 7000xg/rcf and 250 μL of the clear supernatant was transferred into a KingFisher Microtiter deep-well plate. We then added 5 μL of RNase A, mixed by pipetting, and incubated for 10 min at room temperature. After that, we added 290 μL of AL Buffer, 400 μL of HDQ Binding bugger, and 20 μL of Mag-Bind particles HDQ. The plate was then placed into the KingFisher Flex instrument. The subsequent plates in the KingFisher Flex instrument contained: 600 μL of VHB Wash Buffer, 600 μL of VHB Wash Buffer, 600 μL of SPM Buffer, 500 μL of Nuclease-free Water, and 100 μL of Elution Buffer. After the KingFisher Flex instrument protocol run was finished, we transferred the final elute into separate tubes. The DNA quality and quantity was tested using NanoDrop 2000 Spectophotometer (ThermoFisher Scientific)^50^ and stored at −20°C until further analysis.

#### Microsatellite genotyping

We amplified 11 microsatellites (Table S3) using a poolplex reaction following a published protocol.^51^ Briefly, a PCR reaction contained 10.0 μL of Nuclease free water, 0.5 μL of fluorescently labeled forward primer, 0.5 μL of reverse primer, 12.5 μL of TAQ 2X Master Mix (New England Biolabs), and 1.5 μL of DNA sample. We used an ×1000 touch thermocycler (BioRad) with the annealing temperature of 55.0°C. The samples were then sent to The Center for Applied Genomics at The Hospital for Sick Children for automated fragment analysis. We used Geneious (version 11.0) with the Microsatellites plugin to assign alleles and call genotypes, which were checked manually for errors and miscalls. To distinguish patrilines, for each colony, we first deduced the genotype of the queen and assumed that workers with the same haplotype at the 11 microsatellites were sired by the same drone.^28^^,^^51^

#### CYP9Q genotyping

From each colony, the highest and lowest five patrilines in terms of survival with at least five tested bees were chosen for CYP9Q sequencing. We targeted the protein coding sequence (CDS) of three p450 genes (CYP9Q1, CYP9Q2, and CYP9Q3; Table S4), where each gene contains one exon. The PCR reaction was performed as described above with the annealing temperatures of 59.0°C for CYP9Q1 and CYP9Q3, and 58.0°C for CYP9Q2. The samples were sent to The Center for Applied Genomics at The Hospital for Sick Children for sanger sequencing and aligned to reference sequences for these genes from the published honeybee genome using the Geneious (Ver. 11.0) software. CYP9Q1 sequences aligned from nucleotide 172 to 1580 of XM_006562301.3 (CDS: 74-1606), CYP9Q2 sequences aligned from 226 to 1739 of XM_392000.7 (CDS: 172-1770), and CYP9Q3 sequences aligned from 173 to 1567 of XM_006562300.3 (CDS: 85-1638). The coding sequences were translated into amino acid sequences in order to detect non-synonymous nucleotide changes.

#### Identification of SRS and catalytic sites

The six conserved hypothetical P450 substrate recognition sites (SRS)^52^ were identified in all haplotypes of CYP9Q1 and CYP9Q3 by aligning the full amino acid sequences to other insect P450s. Initial sequence and secondary structure alignments carried out utilizing Phyre2^53^ were performed with the reference haplotypes (i.e., the haplotype present in the *A. mellifera* official genome assembly, version Amel_HAv3.1). Preliminary models were built with Phyre2 and used to predict potential catalytic sites using the Catalytic Site Atlas 2.0.^54^ For the CYP9Q1 preliminary model, the catalytic sites identified were Asp307 and Thr308 (both falling in the I-helix), and Phe443 and Cys450 (both in the heme binding region). For the CYP9Q3 preliminary model, the predicted catalytic residues were Ser310 and Ile311 (in the I-helix), and Phe444 and Cys451 (in the heme binding region). The predicted catalytic sites on each of the models correspond to either the conserved site hypothesized to be the important in dioxygen activation during catalytic turnover in the I-helix or in the heme binding region, with Cys450/Cys451 being the residue that directly ligates with heme.

#### Effects of individual mutations

To predict the propensity of a mutation in CYP9Q1 or CYP9Q3 to be associated with reduced survival, we used the SuSPect software,^55^ a predictive software trained with human sequences, 3D homology-based structures, and polymorphisms associated with disease. The result is a matrix of scores that indicate the mutational sensitivity of each residue in the P450 with the reference haplotype as starting point and considering substitutions with each of the 20 other canonical amino acids. Individual mutational effects on the CYP9Qs activities were also evaluated by substituting each of the mutations found in the haplotypes with Missense3D, which considers a total of 17 types of known possible structural damage due to individual amino acid changes.^56^

#### In-silico docking

According to Phyre2, among the CYP enzymes with known crystal structures, the mammalian CYP3A4 has the highest identity to CYP9Q1 and CYP9Q3 (27.4% and 26.7% respectively); hence, the crystal structure of CYP3A4 (PDB ID: 1TQN)^39^ was used as a template to build homology-based models for all the CYP9Q haplotypes using the MOE environment (Chemical Computing Group Inc., Montreal, Canada).

After homology-directed modeling, each of the final energy-minimized structures for each haplotype of CYP9Q1 and CYP9Q3 had phi-psi pairs of at least 76% residues lying in the core regions, and at least 16% residues in other allowed regions, such that 94% of the protein for each of the models were in allowed regions. None of the residues initially predicted to be in the SRS regions or in the catalytic sites initially predicted by Phyre2 fell in the outlier region of the phi-psi plot, indicating that models are of good quality.

#### Dissections and RNA extractions

For Experiment 2, we chose to dissect bees from three of the most tolerant and three of the least tolerant patrilines that had at least five tested bees each. Frozen bees were placed in 1 mL of chilled RNAlater (Invitrogen) and thawed over ice. We performed dissections of the brain, Malpighian tubules, and ventriculus and kept them at −80°C until RNA could be extracted. RNA extractions were completed using the miRNeasy Mini kit (Qiagen). Briefly, we added 700 μL of Qiazol lysis reagent to the tissues and homogenized the solution using a pipette. The protocol provided by the manufacturer was then followed in order to extract the RNA. We added 140 μL of chloroform to the mixture and shook the tubes before allowing them to sit at room temperature for 5 min. We then spun the mixture at 12,000 g for 15 min, and 1.5 volumes of 100% ethanol was added to the upper phase. Once a precipitate had formed, we ran the mixture through an RNeasy spin column. 700 μL of buffer RWT was then added, and once again run through the column, after which 500 μL of buffer RPE was run through the column twice before drying the membrane. Then, 30 μL of RNA-free water was run through the column. RNA quality and quantity were assessed using a NanoDrop ND-1000 UV-Vis.

#### RNAseq and bioinformatic analysis

The ventriculus, brains, and Malpighian tubules of 5 individual bees from each patriline were pooled separately based on their organ (e,g., all the brains of a patriline were pooled together). We had a total of 3 tolerant patrilines, and 3 susceptible patrilines and 18 pools in total. Pooling five bees per patriline increases the power to detect differentially expressed genes with low and medium abundance level.^57^ We focused on a single colony with the highest level of variation in patriline survival, which theoretically would provide the highest variation in gene expression, although this would reduce the generality of our findings.^58^ To ensure equal representation for each bee, we added 300 ng of RNA to each pool (1500 ng/5 bees). Samples were then kept at −80°C. Library preparation and 100 bp pair-end sequencing using the Illumina NovaSeq 6000 S4 were carried out by Genome Quebec’s sequencing facility (Montreal, QC). On average, we sequenced 233 million reads from each sample.

The reads were trimmed using Trimmomatic,^59^ removing adapters and low-quality bases, filtering out reads less than 50 base pairs. Reads were then aligned and indexed against the *A. mellifera* genome (Amel_4.5) using STAR.^60^ Alignment quality was assessed using MultiQC.^61^ HtSeq^62^ was used to count the aligned reads. Differential Expression analysis was carried out using EdgeR.^63^ We used a Benjamini adjustment^64^ of raw p values, and genes with a False Discovery rate (FDR) of <0.05 were considered to be differentially expressed.

#### GO analysis

We used ShinyGO for enriched functional analysis^65^ of Gene Ontology (GO) Processes and Kyoto Encyclopedia of Genes and Genomes (KEGG)^66^ categories, using a false discovery rate cut off of 0.05.

### Quantification and statistical analysis

Statistical analysis was performed using R.^67^ Effect of colony and patriline on survival was performed using the Pearson’s Chi-squared test.^68^ Due to some patrilines having a low number of workers tested, the test was also carried out with the p value computed by a Monte Carlo simulation^69^ with 5000 replicates. Because the two tests yielded similar p values, we report only the former.

The combined effects of colony and patriline were evaluated using a generalized linear model (GLM) with a binomial family structure, where patrilines were nested within colony. Broad sense heritability (*H*^2^) of a phenotype can be estimated by contrasting the phenotypic variance that is explained by patriline relative to the total phenotypic variance. For haplo-diploid organisms, *H*^2^ is twice the patriline variance divided by total phenotypic variance.^25^ Because we used a GLM with a binomial family structure, instead of variance explained, we used deviance explained or adjusted *D*^*2*^ to estimate *H*^*2*^.^70^

The effects of CYP9Q haplotypes on survival were analyzed using a binomial GLM weighed with number of bees run per patriline, with haplotypes nested within colony and analysis of deviance, type 2 for unbalanced design.^70^^,^^71^

We examined how CYP9Q haplotypes were associated with survival rate by performing a classification tree analysis, which iteratively selects the attribute (gene) and value (haplotype) that can split the data into two groups (survived or perished after 24 h exposure), while minimizing the variability within group and maximizing between group contrast,^72^ as implemented in the R package ‘rpart’^73^ To prune the tree we chose the optimal complexity parameter (CP) value with the minimum cross-validated error. Visualization of the tree was performed using the ‘rpart.plot’ package.^74^

## Data Availability

• The raw DNA and RNA data have been deposited at National Center for Biotechnology Information and are publicly available as of the date of publication. Accession numbers are listed in the key resources table. • This paper does not report original code. • Any additional information required to reanalyze the data reported in this paper is available from the lead contact upon request.
