# Supplementary material for: Genetics of tolerance in honeybees to the neonicotinoid clothianidin
Source: iScience. 2023 Feb 2;26(3):106084. doi: 10.1016/j.isci.2023.106084 (PMC9947305; doi:10.1016/j.isci.2023.106084)
Supplement: Document S1. Figures S1–S3 and Tables S1–S4 [file mmc1.pdf]

iScience, Volume *26*

Supplemental information

Genetics of tolerance in honeybees to the neonicotinoid clothianidin

Nadejda Tsvetkov, Simran Bahia, Bernarda Calla, May R. Berenbaum, and Amro Zayed

```
NCBI_CYP9Q1    MDYLQLGLTLLAILVAVYYLSTRNHKLLKRHGIVHIPPTPLFGNLGPLVRRKCHMEDVIQRVYDLDPDA 69
Haplotype_C    ---------------------------------VHIPPTPLFGNLGPLVRRKCHMEDVIQRVYDLDPDA
Haplotype_A    ---------------------------------VHIPPTPLFGNLGPLVRRKCHMEDVIERVYDLDPDA
Haplotype_F    ---------------------------------VHIPPTPLFGNLGPLVRRKCHMEDVIQRVYDLDPDA
Haplotype_D    ---------------------------------VHIPPTPLFGNLGPLVRRKCHMEDVIQRVYDLDPDA
Haplotype_B    ---------------------------------VHIPPTPLFGNLGPLVRRKCHMEDVIQRVYDLDPDA
Haplotype_E    ---------------------------------VHIPPTPLFGNLGPLVRRKCHMEDVIQRVYDLDPDA

NCBI_CYP9Q1    RYVGMYEFTTPLIIIRDPELIKTIGVKEITNFTNHRPFVDVGVDPMLGEVLFAMQGDRWREHRTMLTTL 138
Haplotype_C    RYVGMYEFTTPLIIIRDPELIKTIGVKEITNFTNHRPFVDVGVDPMLGEVLFAMQGDRWREHRTMLTTL
Haplotype_A    RYVGMYEFTTPLIIIRDPELIKTIGVKEITNFTNHRPFVDVGVDPMLGEVLFAMQGDRWREHRTMLTTL
Haplotype_F    RYVGMYEFTTPLIIIRDSELIKTIGVKEITNFTNHRPFVDVGVDPMLGEVLFAMQGDRWREHRTMLTTL
Haplotype_D    RYVGMYEFTTPLIIIRDPELIKTIGVKEITNFTNHRPFVDVGVDPMLGEVLFAMQGDRWREHRTMLTTL
Haplotype_B    RYVGMYEFTTPLIIIRDPELIKTIGVKEITNFTNHRPFVDVGVDPMLGEVLFAMQGDRWREHRTMLTTL
Haplotype_E    RYVGMYEFTTPLIIIRDPELIKTIGVKEITNFTNHRPFVDVGVDPMLGEVLFAMQGDRWREHRTMLTTL

NCBI_CYP9Q1    FTSSKIKSMFVLMSDCAKRFADYLSKVEREIELKSVLTRYTNDVIARCVYGVSVDSVNEPENIFYRYGQ 207
Haplotype_C    FTSSKIKSMFVLMSDCAKRFADYLSKVEREIELKSVLTRYTNDVIARCVYGVSVDSVNEPENIFYRYGQ
Haplotype_A    FTSSKIKSMFVLMSDCAKRFADYLSKVEREIELKSVLTRYTNDVIARCVYGVSVDSVNEPENIFYRYGQ
Haplotype_F    FTSSKIKSMFVLMSDCAKRFADYLSKVEREIELKSVLTRYTNDVIARCVYGVSVDSVNEPENIFYRYGQ
Haplotype_D    FTSSKIKSMFVLMSDCAKRFADYLSKVEREIELKSVLTRYTNDVIARCVYGVSVDSVNEPENIFYRYGQ
Haplotype_B    FTSSKIKSMFVLMSDCAKRFADYLSKVEREIELKSVLTRYTNDVIARCVYGVSVDSVNEPENIFYRYGQ
Haplotype_E    FTSSKIKSMFVLMSDCAKRFADYLSKVEREIELKSVLTRYTNDVIARCVYGVSVDSVNEPENIFYRYGQ

NCBI_CYP9Q1    VASQLSTFKQNLMIFVHRNSPRLARLFNLKILPVHIEKFFHRLVMDTIETRRREGVHGLDMLQQLMDMQ 276
Haplotype_C    VASQLSTFKQNLMIFVHRNSPRLARLFNLKILPVHIEKFFHRLVMDTIETRRREGVHGLDMLQQLMDMQ
Haplotype_A    VASQLSTFKQNLMIFVHRNSPRLARLFNLKILPVHIEKFFHRLVMDTIETRRREGVHGLDMLQQLMDMQ
Haplotype_F    VASQLSTFKQNLMIFVHRNSPRLARLFNLKILPVHIEKFFHRLVMDTIETRRREGVHGLDMLQQLMDMQ
Haplotype_D    VASQLATFKQNLMIFVHRNSPRLARLFNLKILPVHIEKFFHRLVMDTIETRRREGVHGLDMLQQLMDMQ
Haplotype_B    VASQLATFKQNLMIFVHRNSPRLARLFNLKILPVHIEKFFHRLVVDTIEARRREGVHGLDMLQQLMDMQ
Haplotype_E    VASQLATFKQNLMIFVHRNSPRLARLFNLKILPVHIEKFFHRLVVDTIEARRRECTGWTCCNN------

NCBI_CYP9Q1    SRRKESEEGKRGMTVTDIANHAFSFFFGSVDTMATQISLISHMLAVNPDVQQRLQEEIDEVLSASEDKQ 345
Haplotype_C    SRRKESEEGKRGMTVTDIANHAFSFFFGSVDTMATQISLISHMLAVNPDVQQRLQEEIDEVLSASEDKQ
Haplotype_A    SRRKESEEGKRGMTVTDIANHAFSFFFGSVDTMATQISLISHMLAVNPDVQQRLQEEIDEVLSASEDKQ
Haplotype_F    SRRKESEEGKRGMTVTDIANHAFSFFFGSVDTMATQISLISHMLAVNPDVQQRLQEEIDEVLSASEDKQ
Haplotype_D    SRRKESEEGKRGMTVTDIANHAFSFFFGSVDTMATQISLISHMLAVNPDVQQRLQEEIDEVLSASEDKQ
Haplotype_B    SRRKESEEGKRGMTVTDIANHAFSFFFGSVDTMATQISLISHMLAVNPDVQQRLQEEIDEVLSASEDKQ
Haplotype_E    ---------------------------------------------------------------------

NCBI_CYP9Q1    VGYDVIQEMKYLDAVMSEAMRYHPILLFVDRVCGETFELPPALPGARPFKLERGMNIWFPVKAIHHDPK 414
Haplotype_C    VGYDVIQEMKYLDAVMSEAMRYHPILLFVDRVCGETFELPPALPGARPFKLERGMNIWFPVKAIHHDPK
Haplotype_A    VGYDVIQEMKYLDAVMSEAMRYHPILLFVDRVCGETFELPPALPGARPFKLERGMNIWFPVKAIHHDPK
Haplotype_F    VGYDVIQEMKYLDAVMSEAMRYHPILLFVDRVCGETFELPPALPGARPFKLERGMNIWFPVKAIHHDPK
Haplotype_D    VGYDVIQEMKYLDAVMSEAMRYHPILLFVDRVCGETFELPPALPGARPFKLERGMNIWFPVKAIHHDPK
Haplotype_B    VGYDVIQEMKYLDAVMSEAMRYHPILLFVDRVCGETFELPPALPGARPFKLERGMNIWFPVKAIHHDPK
Haplotype_E    ---------------------------------------------------------------------

NCBI_CYP9Q1    YFENPDRFDPDRFLRDGKGIASSGAYMPFGMGPRKCIGSRFALTEMKILLFNILAKCSFKVGSKTMVPL 483
Haplotype_C    YFENPDRFDPDRFLRDGKGIASSGAYMPFGMGPRKCIGSRFALTEMKILLFNILAKCSFKVGSKTMVPL
Haplotype_A    YFENPDRFDPDRFLRDGKGIASSGAYMPFGMGPRKCIGSRFALTEMKILLFNILAKCSFKVGSKTMVPL
Haplotype_F    YFENPDRFDPDRFLRDGKGIASSGAYMPFGMGPRKCIGSRFALTEMKILLFNILAKCSFKVGSKTMVPL
Haplotype_D    YFENPDRFDPDRFLRDGKGIASSGAYMPFGMGPRKCIGSRFALTEMKILLFNILAKCSFKVGSKTMVPL
Haplotype_B    YFENPDRFDPDRFLRDGKGIASSGAYMPFGMGPRKCIGSRFALTEMKILLFNILAKCSFKVGSKTMVPL
Haplotype_E    ---------------------------------------------------------------------

NCBI_CYP9Q1    KFKEGVFNPVAKNGFWLKIERRENSCC 510
Haplotype_C    KFKEGVFNPVAKNGFWLKI--------
Haplotype_A    KFKEGVFNPVAKNGFWLKI--------
Haplotype_F    KFKEGVFNPVAKNGFWLKI--------
Haplotype_D    KFKEGVFNPVAKNGFWLKI--------
Haplotype_B    KFKEGVFNPVAKNGFWLKI--------
Haplotype_E    ---------------------------
```

Figure S1. CYP9Q1 haplotypes. Related to Figure 3 and Table 1. Putative substrate recognition sites (SRS) are underlined based on Schuler and Berenbaum[1] . Bolded amino acid residues represent the sites of non-synonymous mutations. Haplotypes with a grey background were found to be associated with a lower survival rate.

```
NCBI_CYP9Q2     MEFLSLALVLAAISIIAYYYCFVRKNFNLFQEHGILHVPPSPLVGNFGPLIRGKENVHDTIQRIYNIHP69
Haplotype_H     ------------------YYCFVRKNFNLFQEHGILHVPPSPLVGNFGPLIRGKENVHDTIQRIYNIHP
Haplotype_J     ------------------YYCFVRKNFNLFQEHGILHVPPSPLVGNFGPLIRGKENVHDTIQRIYNIHP
Haplotype_I     ------------------YYCFVRKNFNLFQEHGILHVPPSPLVGNFGPLIRGKENVHDTIQRIYNIHP
Haplotype_G     ------------------YYCFVRKNFNLFQEHGILHVPPSPLVGNFGPLIRGKENVHDTIQRIYNIHP

NCBI_CYP9Q2     DAKYVGIFEFLTPVIMIRDLDLIKSITMKNFDQFPDHRPMFCKSVDPMLGEMLFIMDGERWKEHRNMLS138
Haplotype_H     DAKYVGIFEFLTPVIMIRDLDLIKSITMKNFDQFPDHRPMFCKSVDPMLGEMLFIMDGERWKEHRNMLS
Haplotype_J     DAKYVGIFEFLTPVIMIRDLDLIKSITMKNFDQFPDHRPMFCKSVDPMLGEMLFIMDGERWKEHRNMLS
Haplotype_I     DAKYVGIFEFLTPVIMIRDLDLIKSITMKNFDQFPDHRPMFCKSVDPMLGEMLFIMDGERWKEHRNMLS
Haplotype_G     DAKYVGIFEFLTPVIMIRDLDLIKSITMKNFDQFPDHRPMFCKSVDPMLGEMLFIMDGERWKEHRNMLS

NCBI_CYP9Q2     PTFTSSKIKTMFVHMSECAKRFAHHLSKLPEKDRETEMKALLTRYTNDVIAACIYGVNVDSIKEPRNVF207
Haplotype_H     PTFTSSKIKTMFVHMSECAKRFAHHLSKLPEKDRETEMKALLTRYTNDVIAACIYGVNVDSIKEPRNVF
Haplotype_J     PTFTSSKIKTMFVHMSECAKRFAHHLSKLPEKDRETEMKALLTRYTNDVIAACIYGVNVDSIKEPRNVF
Haplotype_I     PTFTSSKIKTMFVHMSECAKRFAHHLSKLPEKDRETEMKALLTRYTNDVIAACIYGVNVDSIKEPRNVF
Haplotype_G     PTFTSSKIKTMFVHMSECAKRFAHHLSKLPEKDRETEMKALLTRYTNDVIAACIYGVNVDSIKEPRNVF

NCBI_CYP9Q2     YMYGRVGATLIGLKKNLKIMVHRNMPWLANLLRLNILERHIAKFFTDLVVETVEERERNGTTNSDLIQL276
Haplotype_H     YMYGRVGATLIGLKKNLKIMVHRNMPWLANLLRLNILERHIAKFFTDLVVETVEERERNGTTNSDLIQL
Haplotype_J     YMYGRVGATLIGLKKNLKIMVHRNMPWLANLLRLNILERHVAKFFTDLVVETVEERERNGTTNSDLIQL
Haplotype_I     YMYGRVGATLIGLKKNLKIMVHRNMPWLANLLRLNILERHVAKFFTDLVVETVEERERNGTTNSDLIQL
Haplotype_G     YMYGRVGATLIGLKKNLKIMVHRNMPWLANLLRLNILERHIAKFFTDLVVETVEERERNGTTNSDLIQL

NCBI_CYP9Q2     MMDTRNKKESGKKNLTVQNMANHAFSFFFGGFDTVSSQTCVLLHMLVENPEVQQRLQQEIDETLESNNG345
Haplotype_H     MMDTRNKKESGKKNLTVQNMANHAFSFFFGGFDTVSSQTCVLLHMLVENPEVQQRLQQEIDETLESNNG
Haplotype_J     MMDTRNKKESGKKNLTVQNMANHAFSFFFGGFDTVSSQTCVLLHMLVENPEVQQRLQQEIDETLESNNG
Haplotype_I     MMDTRNKKESGKKNLTVQNMANHAFSFFFGGFDTVSSQTCVLLHMLVENPEVQQRLQQEIDETLESNNG
Haplotype_G     MMDTRNKKESGKKNLTVQNMANHAFSFFFGGFDTVSSQTCVLLHMLVENPEVQQRLQQEIDETLESNNG

NCBI_CYP9Q2     QLSYDVIQEMRYLDAVINEILRLHPIAVFIDRMCVKSFELPPALPGDVPFTVKPGMNVWIPVKAIHHDP414
Haplotype_H     QLSYDVIQEMRYLDAVINEILRLHPIAVFIDRMCVKSFELPPALPGDVPFTVKPGMNVWIPVKAIHHDP
Haplotype_J     QLSYDVIQEMRYLDAVINEILRLHPIAVFIDRMCVKSFELPPALPGDVPFTVKPGMNVWIPVKAIHHDP
Haplotype_I     QLSYDVIQEMRYLDAVINEILRLHPIAVFIDRMCVKSFELPPALPGDVPFTVKPGMNVWIPVKAIHHDP
Haplotype_G     QLSYDVIQEMRYLDAVINEILRLHPIAVFIDRMCVKSFELPPALPGDVPFTVKPGMNVWIPVKAIHHDP

NCBI_CYP9Q2     RYYDEPEKFKPERFLDNGKNIIGSGAYFPFGIGPRICIGNRFALIEMKVLVCHILAVCDIKAGARTGIP483
Haplotype_H     RYYDEPEKFKPERFLDNGKNIIGSGAYFPFGIGPRICIGNRFALIEMKVLVCYILAVCDIKAGARTGIP
Haplotype_J     RYYDEPEKFKPERFLDNGKNIIGSGAYFPFGIGPRICIGNRFALIEMKVLVCYILAVCDIKAGARTGIP
Haplotype_I     RYYDEPEKFKPERFLDNGKNIIGSGAYFPFGIGPRICIGNRFALIEMKVLVCHILAVCDIKAGARTGIP
Haplotype_G     RYYDEPEKFKPERFLDNGKNIIGSGAYFPFGIGPRICIGNRFALIEMKVLVCHILAVCDIKAGARTGIP

NCBI_CYP9Q2     LEFEKGVFNATAKTGFWLKIEPRKYSYHSGQINGLVNNHVINGACKTGI532
Haplotype_H     LEFEKGVFNATAKTGFWLKIEPRKYSYHSGQINGLVNNH----------
Haplotype_J     LEFEKGVFNATAKTGFWLKIEPRKYSYHSGQINGLVNNH----------
Haplotype_I     LEFEKGVFNATAKTGFWLKIEPRKYSYHSGQINGLVNNH----------
Haplotype_G     LEFEKGVFNATAKTGFWLKIEPRKYSYHSGQINGLVNNH----------
```

Figure S2. CYP9Q2 haplotypes. Related to Figure 3. Putative substrate recognition sites (SRS) are underlined and based on Schuler and Berenbaum[1]. Bolded amino acid residues represent the sites of non-synonymous mutations.

```
NCBI_CYP9Q3   MDYLTISLSLITVFVAVYYLATRNNDFFKKHGIPHVPPVPFLGNMGSLVRQKSNLHDVIDRTYNLDPGA 69
Haplotype_O   ------------------------------HGIPHVPPVPFLGNMGSLVRQKSNLHDVIDRMYNLDPGA
Haplotype_K   ------------------------------HGIPHVPPVPFLGNMGSLVRQKSNLHDVIDRMYNLDPGA
Haplotype_Q   ------------------------------HGIPHVPPVPFLGNMGSLVRQKSNLHDVIDRTYNLDPGA
Haplotype_M   ------------------------------HGIPHVPPVPFLGNMGSLVRQKSNLHDVIDRMYNLDPGA
Haplotype_N   ------------------------------HGIPHVPPVPFLGNMGSLVRQKSNLHDVIDRMYNLDPGA
Haplotype_L   ------------------------------HGIPHVPPVPFLGNMGSLVRQKSNLHDVIDRMYNLDPGA
Haplotype_P   ------------------------------HGIPHVPPVPFLGNMGSLVRQKSNLHDVIDRTYNLDPGA

NCBI_CYP9Q3   KYVGIYEFTTPIIILRDLDLIKTITMKYLDHFPDHRSFAYEGADPVFGSMLFAMKGERWKEHRNMLTPT 138
Haplotype_O   KYVGIYEFTTPIIILRDLDLIKTITMKYLDHFPDHRSFAYEGADPVFGSMLFAMKGERWKEHRNMLTPT
Haplotype_K   KYVGIYEFTTPIIILRDLDLIKTITMKYLDHFPDHRSFAYEGADPVFGSMLFAMKGERWKEHRNMLTPT
Haplotype_Q   KYVGIYEFTTPIIILRDLDLIKTITMKYLDHFPDHRSFAYEGADPVFGSMLFAMKGERWKEHRNMLTPT
Haplotype_M   KYVGIYEFTTPIIILRDLDLIKTITMKYLDHFPDHRSFAYEGADPVFGSMLFAMKGERWKEHRNMLTPT
Haplotype_N   KYVGIYEFTTPIIILRDLDLIKTITMKYLDHFPDHRSFAYEGADPVFGSMLFAMKGERWKEHRNMLTPT
Haplotype_L   KYVGIYEFTTPIIILRDLDLIKTITMKYLDHFPDHRSFAYEGADPVFGSMLFAMKGERWKEHRNMLTPT
Haplotype_P   KYVGIYEFTTPIIILRDLDLIKTITMKYLDHFPDHRSFAYEGADPVFGSMLFAMKGERWKEHRNMLTPT

NCBI_CYP9Q3   LTSSKIKGMFKLMTECAVRFADFLSVLPENERETEMKALLSRYANDVIASCVYGVSVDSINDPKNIFYV 207
Haplotype_O   LTSSKIKGMFKLMTECAVRFADFLSVLPENERETEMKALLSRYANDVIASCVYGVSVDSINDPKNIFYV
Haplotype_K   LTSSKIKGMFKLMIECAVRFADFLSVLPENERETEMKALLSRYANDVIASCVYGVSVDSINDPKNIFYV
Haplotype_Q   LTSSKIKGMFKLMTECAVRFADFLSVLPENERETEMKALLSRYANDVIASCVYGVSVDSINDPKNIFYV
Haplotype_M   LTSSKIKGMFKLMTECAVRFADFLSVLPENERETEMKALLSRYANDVIASCVYGVSVDSINDPKNIFYV
Haplotype_N   LTSSKIKGMFKLMTECAVRFADFLSVLPENERETEMKALLSRYANDVIASCVYGVSVDSINDPKNIFYV
Haplotype_L   LTSSKIKGMFKLMTECAVRFADFLSVLPENERETEMKALLSRYANDVIASCVYGVSVDSINDPKNIFYV
Haplotype_P   LTSSKIKGMFKLMTECAVRFADFLSVLPENERETEMKALLSRYANDVIASCVYGVSVDSINDPKNIFYV

NCBI_CYP9Q3   YGRRGTNVVGLKKSMFVLIHRNMPWLAKLFGLRFLEKHVQKFFYDLVYETIESREKLGTNRSDVLQLLM 276
Haplotype_O   YGRRGTNVVGLKKSMFVLIHRNMPWLAKLFGLRFLEKHVQKFFYDLVYETIESREKLGTNRSDVLQLLM
Haplotype_K   YGRRGTNVVGLKKSMFVLIHRNMPWLAKLFGLRFLEKHVQKFFYDLVYETIESREKLGTNRSDVLQLLM
Haplotype_Q   YGRRGTNVVGLKKSMFVLIHRNMPWLAKLFGLRFLEKHVQKFFYDLVYETIESREKLGTNRSDVLQLLM
Haplotype_M   YGRRGTNVVGLKKSMFVLIHRNMPWLAKLFDLRFLEKHVQKFFYDLVYETIESREKLGTNRSDVLQLLM
Haplotype_N   YGRRGTNVVGLKKSMFVLIHRNMPWLAKLFRLRFLEKHVQKFFYDLVYETIESREKLGTNRSDVLQLLM
Haplotype_L   YGRRGTNVVGLKKSMFVLIHRNMPWLAKLFGLRFLEKHVQKFFYDLVYETIESREKLGTNRSDVLQLLM
Haplotype_P   YGRRGTNVVGLKKSMFVLIHRNMPWLAKLFGLRFLEKHVQKFFYDLVYETIESREKLGTNRSDVLQLLM

NCBI_CYP9Q3   DIRDKANSSGKMTTMTVENVAIHAFTFFFGGFDSITSVTTLLTQMLAEHPDVQARLQQEIDETLRSNDG 345
Haplotype_O   DIRDKANSSGKMTTMTVENVAIHAFTFFFGGFDSITSVTTLLTQMLAEHPDVQARLQQEIDETLRSNDG
Haplotype_K   DIRDKANSSGKMTTMTVENVAIHAFTFFFGGFDSITSVTTLLTQMLAEHPDVQARLQQEIDETLRSNDG
Haplotype_Q   DIRDKANSSGKMTTMTVENVAIHAFTFFFGGFDSITSVTTLLTQMLAEHPDVQARLQQEIDETLRSNDG
Haplotype_M   DIRDKANSSGKMTTMTVENVAIHAFTFFFGGFDSITSVTTILTQMLAEHPDVQARLQQEIDETLRSNDG
Haplotype_N   DIRDKANSSGKMTTMTVENVAIHAFTFFFGGFDSITSVTTILTQMLAEHPDVQARLQQEIDETLRSNDG
Haplotype_L   DIRDKANSSGKTTTMTVENVAIHAFTFFFGGFDSITSVTTLLTQMLAEHPDVQARLQQEIDETLRSNDG
Haplotype_P   DIRDKANSSGKMTTMTVENVAIHAF-FFFGGFDSITSVTTLLTQMLAEHPDVQARLQQEIDETLRSNDG

NCBI_CYP9Q3   VLTYDAVHGMKYMDAVINETMRFCPVLPFLDRMCVESFQLPAPVPGGQPFTLRPGMNVWIPLAAIGRDP 414
Haplotype_O   VLTYDAVHGMKYMDAVINETMRFCPVLPFLDRMCVESFQLPAPVPGGQPFTLRPGMNVWIPLAAIGRDP
Haplotype_K   VLTYDAVHGMKYMDAVINETMRFCPVLPFLDRMCVESFQLPAPVPGGQPFTLRPGMNVWIPLAAIGRDP
Haplotype_Q   VLTYDAVHGMKYMDAVINETMRFCPVLPFLDRMCVESFQLPAPVPGGQPFTLRPGMNVWIPLAAIGRDP
Haplotype_M   VLTYDAVHGMKYMDAVINETMRFCPVLPFLDRMCAESFQLPPPVPGGQPFTLRPGMNVWIPLAAIGRDP
Haplotype_N   VLTYDAVHGMKYMDAVINETMRFCPVLPFLDRMCAESFQLPPPVPGGQPFTLRPGMNVWIPLAAIGRDP
Haplotype_L   VLTYDAVHGMKYMDAVINETMRFCPVLPFLDRMCAESFQLPAPVPGGQPFTLRPGMNVWIPLAAIGRDP
Haplotype_P   VLTYDAVHGMKYMDAVINETMRFCPVLPFLDRMCVESFQLPAPVPGGQPFTLRPGMNVWIPLAAIGRDP

NCBI_CYP9Q3   EYFEDPDKFDPDRFLNPEAGIKNSGAHFPFGLGQRKCIGERFAMMEMKVLLCYVLAACNVRIGSKTTVP 483
Haplotype_O   EYFEDPDKFDPDRFLNPEAGIKNSGAHFPFGLGQRKCIGERFAMMEMKVLLCYVLAACNVRIGSKTTVP
Haplotype_K   EYFEDPDKFDPDRFLNPEAGIKNSGAHFPFGLGQRKCIGERFAMMEMKVLLCYVLAACNVRIGSKTTVP
Haplotype_Q   EYFEDPDKFDPDRFLNPEAGIKNSGAHFPFGLGQRKCIGERFAMMEMKVLLCYVLAACNVRIGSKTTVP
Haplotype_M   EYFEDPDKFDPDRFLNPEAGIKNSGAHFPFGLGQRKCIGERFAMMEMKVLLCYVLAACNVRIGSKTTVP
Haplotype_N   EYFEDPDKFDPDRFLNPEAGIKNSGAHFPFGLGQRKCIGERFAMMEMKVLLCYVLAACNVRIGSKTTVP
Haplotype_L   EYFEDPDKFDPDRFLNPEAGIKNSGAHFPFGLGQRKCIGERFAMMEMKVLLCYVLAACNVRIGSKTTVP
Haplotype_P   EYFEDPDKFDPDRFLNPEAGIKNSGAHFPFGLGQRKCIGERFAMMEMKVLLCYVLAACNVRIGSKTTVP

NCBI_CYP9Q3   MKLEKGLINANVKGGFWLKIEPRKVTYYNSSRSN 517
Haplotype_O   MKLEKGLINAN-----------------------
Haplotype_K   MKLEKGLINAN-----------------------
Haplotype_Q   MKLEKGLINAN-----------------------
Haplotype_M   MKLEKGLINAN-----------------------
Haplotype_N   MKLEKGLINAN-----------------------
Haplotype_L   MKLEKGLINAN-----------------------
Haplotype_P   MKLEKGLINAN-----------------------
```

Figure S3. CYP9Q3 haplotypes. Related to Figure 3 and Table 1. Putative substrate recognition sites (SRS) are underlined and based on Schuler and Berenbaum[1]. Bolded amino acid residues represent the sites of non-synonymous mutations. Haplotypes with grey a background were found to be associated with a lower survival rate.

Table S1. List of DEGs in the Malpighian tubules between most tolerant and least tolerant patrilines exposed to 4.27ppb of clothianidin. Related to Figure 2.

Gene	logFC	FDR	Protein
XM_026445507.1	-4.30	0.001	dynein beta chain, ciliary
XR_001703097.2	-4.24	0.001	uncharacterized LOC107964495
XM_392226.7	-3.84	0.040	calmodulin
XM_006563260.2	-3.81	0.006	Apis mellifera fructose-bisphosphate aldolase (LOC550785), transcript variant X2, mRNA
XM_006564738.3	-3.76	0.000	uncharacterized LOC412969
XM_006559239.3	-3.49	0.001	protein neuralized
XM_026444499.1	-3.29	0.025	uncharacterized LOC410363
XM_016912656.2	-3.16	0.001	transcription factor Sox-13
XM_026445881.1	-3.12	0.012	Apis mellifera uncharacterized LOC100577769
XR_001702612.2	-3.02	0.001	uncharacterized LOC107964233
XM_016911272.2	-3.02	0.004	Allatostatin C receptor
XM_394827.6	-2.99	0.000	lipase 3
XM_392043.7	-2.90	0.000	endothelin-converting enzyme homolog [Apis mellifera (honey bee)
XM_003249830.4	-2.84	0.010	Apis mellifera uncharacterized LOC100577110
XM_026440879.1	-2.81	0.000	LOC726309 protein artichoke
XR_001703293.2	-2.81	0.004	uncharacterized LOC107964569
XR_408967.3	-2.77	0.000	uncharacterized LOC102656563
XM_006565484.3	-2.77	0.000	aminopeptidase N
XM_016913279.2	-2.75	0.000	vanin-like protein 1
XM_016913467.2	-2.71	0.030	uncharacterized LOC413052
XM_016914011.2	-2.68	0.033	probable beta-hexosaminidase fdl
XM_006572315.3	-2.66	0.032	adenylate kinase isoenzyme 5
XM_006561437.3	-2.61	0.000	protein slit [Apis mellifera (honey bee)]
XM_394067.6	-2.59	0.001	semaphorin-5A
XM_026444698.1	-2.56	0.000	transcription factor Sox-2
XR_003306397.1	-2.48	0.018	uncharacterized LOC113219352
NM_001011636.1	-2.48	0.000	Fabp FABP-like protein
XM_003251822.4	-2.45	0.000	uncharacterized LOC100577161
XM_016916443.2	-2.44	0.011	Apis mellifera zinc finger BED domain-containing protein 1
XR_003306009.1	-2.44	0.000	uncharacterized LOC107965414
XM_006557650.3	-2.43	0.000	uncharacterized LOC102654685
XM_006565481.3	-2.40	0.000	aminopeptidase N
XM_026442484.1	-2.40	0.017	mitochondrial uncoupling protein 2
NR_039429.1	-2.38	0.015	Apis mellifera microRNA mir-1175
XM_006564914.3	-2.37	0.032	facilitated trehalose transporter Tret1

XR_003306097.1	-2.33	0.000	uncharacterized LOC107965483
XM_393060.7	-2.29	0.000	pancreatic triacylglycerol lipase
XM_026439414.1	-2.27	0.022	cGMP-dependent protein kinase, isozyme 1
XM_003249136.4	-2.25	0.049	uncharacterized LOC100578100
NM_001013361.1	-2.24	0.000	18-w 18-wheeler
XM_026446208.1	-2.21	0.023	ionotropic receptor 25a
XM_026444170.1	-2.18	0.046	insulin-like growth factor 2 mRNA-binding protein 1
XM_006565067.3	-2.17	0.000	putative polypeptide N-acetylgalactosaminyltransferase 9 [
XM_003250107.4	-2.15	0.003	short neuropeptide F
XM_003250921.4	-2.14	0.000	uncharacterized LOC100578548
XM_001120112.5	-2.13	0.004	serine protease 53
XM_026443683.1	-2.11	0.003	mucin-17-like
XM_026439740.1	-2.10	0.000	uncharacterized LOC100578706
XM_006559465.3	-2.09	0.033	uncharacterized LOC100577488
XM_623673.6	-2.09	0.000	putative serine protease K12H4.7
XM_006561640.3	-2.07	0.049	homeobox protein Nkx-2.8
XM_026442221.1	-2.03	0.033	mast/stem cell growth factor receptor Kit
NM_001327967.1	-2.01	0.003	juvenile hormone acid O-methyltransferase
XM_016910864.2	-2.01	0.001	plastin-2
XR_407492.3	-2.00	0.033	uncharacterized LOC100578394
XM_001121077.5	-1.98	0.019	chymotrypsin inhibitor
XM_003251239.4	-1.97	0.010	Apis mellifera uncharacterized LOC100578412
NM_001134949.1	-1.97	0.006	Apis mellifera glucosamine-fructose-6-phosphate aminotransferase 2 (Gfat2), mRNA
XM_003250751.4	-1.96	0.000	uncharacterized LOC100578731
XM_026444761.1	-1.95	0.000	cationic amino acid transporter 4
XM_392630.6	-1.95	0.000	uncharacterized LOC409105
XR_120331.4	-1.94	0.005	uncharacterized LOC100576585
XM_394855.6	-1.91	0.017	Apis mellifera caspase-3
XM_016913943.2	-1.88	0.000	NPC intracellular cholesterol transporter 1 homolog 1b
XR_119746.4	-1.88	0.000	uncharacterized LOC100576397
XR_003305949.1	-1.83	0.003	uncharacterized LOC113219194
XM_001121102.5	-1.83	0.040	ninjurin-2
XR_001705202.1	-1.83	0.031	uncharacterized LOC107965347
XM_006561800.3	-1.80	0.003	beta-hexosaminidase subunit beta
XM_016914797.2	-1.79	0.004	uncharacterized LOC408320
XM_003251355.4	-1.78	0.017	Apis mellifera uncharacterized LOC100577128
XM_026445002.1	-1.78	0.004	growth/differentiation factor 8
XM_026444640.1	-1.77	0.000	uncharacterized LOC107965746

XM_001121910.4	-1.71	0.017	transcription factor Sox-21-B
XM_016916502.2	-1.71	0.012	Apis mellifera trypsin
XM_026445397.1	-1.69	0.042	disintegrin and metalloproteinase domain-containing protein 10-like
XM_006566014.3	-1.69	0.003	four and a half LIM domains protein 2
XM_026442934.1	-1.68	0.005	hydrocephalus-inducing protein-like
XM_006560978.3	-1.66	0.028	uncharacterized LOC412774
XM_026442948.1	-1.65	0.033	uncharacterized LOC409465
XM_016911303.2	-1.63	0.007	Apis mellifera uncharacterized LOC107964189
XM_001120425.5	-1.63	0.012	Apis mellifera uncharacterized LOC724536
XM_006562350.3	-1.60	0.018	uncharacterized LOC409057
XM_006570213.3	-1.58	0.033	broad-complex core protein isoforms 1/2/3/4/5
XM_003249633.4	-1.58	0.000	uncharacterized LOC100578090
XM_395905.6	-1.54	0.001	zinc transporter ZIP1
XM_016917454.2	-1.50	0.000	sodium/potassium/calcium exchanger 3
XM_026442378.1	-1.48	0.017	sphingomyelin phosphodiesterase 1
XM_026444746.1	-1.47	0.018	probable cytochrome P450 6a14
XM_006564895.3	-1.44	0.009	Apis mellifera uncharacterized LOC100578813
XR_001702082.2	-1.28	0.003	uncharacterized LOC107964028
XM_026445495.1	-1.26	0.033	PH and SEC7 domain-containing protein 2
XM_026439095.1	-1.26	0.001	uncharacterized LOC408661
XM_003249567.4	-1.24	0.000	lateral signaling target protein 2 homolog
XM_026439235.1	-1.24	0.009	Apis mellifera uncharacterized LOC725588
XM_026444401.1	1.32	0.000	fatty-acid amide hydrolase 2-B

Table S2. Simulated docking parameters for clothianidin in the predicted active pocket of homology-based models of CYP9Q1 and CYP9Q3 haplotypes. Related to Table 1.

Protein	Haplotype	Average distance from clothianidin methyl group to heme after E minimization (Å)	Average distance from heme intermediate to the nearest carbon in the P450 Thr308 (I-Helix) after final minimization (Å)	Final interacting energy
CYP9Q1	D	4.558	4.85	-39.74
CYP9Q1	B	5.834	4.99	-55.54
CYP9Q1	A	6.12	5.34	-32.51
CYP9Q1	C (WT)	6.92	3.86	-43.20
CYP9Q1	F	8.332	4.27	-38.62
CYP9Q3	O	4.854	4.72	-69.264
CYP9Q3	N	4.566	5.02	-35.802
CYP9Q3	K	5.194	5.81	-41.082
CYP9Q3	M	7.572	6.59	-42.284
CYP9Q3	L	8.488	5.21	-45.728
CYP9Q3	Q (WT)	6.730	4.80	-51.884
CYP9Q3	P	9.566	4.95	-29.368

Table S3. The list of primers used to discern the patrilines of the tested worker bees. Related to STAR Methods. F: Forward primer, R: Reverse primer. Italic text denoted the name of the fluorophore dye.

**Loci**	**Primer Sequence (5' – 3')**
HB-SEX-02	F: *HEX*-ACGCATTGAAGGATATTATGA<br>R: AATTTGAACATTCGATCACC
HB-THE-03	F: *FAM*-TAACTGGTCGTCGGTGTT<br>R: CACGTAGAGAATCCCATTGT
AC006	F: *PET*-GATCGTGGAAACCGCGAC<br>R: CACGGCCTCGTAACGGTC
HB-C16-05	F: *NED*-ATTTTATGCGCGTTTCGTA<br>R: CATGGCTCCTCCATTAAATC
HB-C16-01	F: *HEX*-AAAATGCGATTCTAATCTGG<br>R: TTGCCTAAAATGCTTGCTAT
A024	F: *FAM*-CACAAGTTCCAACAATGC<br>R: CACATTGAGGATGAGCG
A107	F: *NED*-CCGTGGGAGGTTTATTGTCG<br>R: CCTTCGTAACGGATGACACC
A007	F: *PET*-GTTAGTGCCCTCCTCTTGC<br>R: CCCTTCCTCTTTCATCTTCC
A079	F: *HEX*-CGAAGGTTGCGGAGTCCTC<br>R: GTCGTCGGACCGATGCG
A113	F: *NED*-CTCGAATCGTGGCGTCC<br>R: CCTGTATTTTGCAACCTCGC
HB-THE-02	F: *FAM*-GGGAAAGATATTAGGGAGGA<br>R: CGACGAAAAATTACAAGGAC

Table S4. The list of primers used to sequence the three CYP9Q genes. Related to STAR Methods. F: Forward primer, R: Reverse primer.

**Gene**	**Primer sequence (5' – 3')**
CYP9Q1_A	F: ACCTGTCCACGAGGAATCAC<br>R: TCTTTCCTCCTCGATTGCAT
CYP9Q1_B	F: GACACGATCGAGACGAGGA<br>R: CAAGAATTCTCCCTGCGTTC
CYP9Q2_A	F: TTATCGTTGGCTCTCGTCCT<br>R: ACCCGATTCCTTCTTGTTCC
CYP9Q2_B	F: CGAGGAACAAGAAGGAATCG<br>R: ACACGCGCCGTTAATAACAT
CYP9Q3_A	F: CGTATTCGTGGCCGTTTATT<br>R: TTCGCCTTGTCCCTTATGTC
CYP9Q3_B	F: CTCGAGAAACACGTGCAAAA<br>R: ACCTTCCTAGGCTCGATCTTC

Supplemental references section

1. Schuler, M.A., and Berenbaum, M.R. (2013). Structure and function of cytochrome P450S in insect adaptation to natural and synthetic toxins: insights gained from molecular modeling. Journal of chemical ecology *39*, 1232-1245.
